# Association between age of first exposure and heavy internet use in a representative sample of 317,443 adolescents from 52 countries

**DOI:** 10.1007/s00787-021-01869-5

**Published:** 2021-09-12

**Authors:** Rubén López-Bueno, Ai Koyanagi, Guillermo Felipe López-Sánchez, Joseph Firth, Lee Smith

**Affiliations:** 1 Department of Physical Medicine and Nursing, University of Zaragoza, 50009 Zaragoza, Spain; 2 Research and Development Unit, Parc Sanitari Sant Joan de Déu, CIBERSAM, ICREA, Barcelona, Spain; 3 Vision and Eye Research Institute, School of Medicine, Faculty of Health, Education, Medicine and Social Care, Anglia Ruskin University, Cambridge, UK; 4 Division of Psychology and Mental Health, Manchester Academic Health Science Centre, University of Manchester, Manchester, UK; 5 Greater Manchester Mental Health NHS Foundation Trust, Manchester Academic Health Science Centre, Manchester, UK; 6 Centre for Health, Performance and Wellbeing, Anglia Ruskin University, Cambridge, UK

**Keywords:** Social media, Video games, Addictions, Mental health, Health habits

## Abstract

Internet usage among adolescents has increased substantially over the last years, concurrently with emerging concerns that an abusive use is associated with detrimental health outcomes. Our objective was to examine the association between age of first exposure and heavy Internet usage in different domains. Data from the 2018 wave of the Programme for International Student Assessment (PISA) were retrieved. This included a total of 317,443 participants (49.2% boys) aged 15 and 16 years from 52 countries. Data from both Internet use and age of first exposure were retrieved and used to conduct metanalyses with random effects. Adolescents reporting an age of first exposure of Internet usage at ≥ 13 years old had the lowest odds for heavy Internet use (> 2 h/day) (reference group: ≤ 9 years) during weekends (odds ratio, 0.41 [95% CI, 0.35–0.48]), weekdays (odds ratio, 0.45 [95% CI, 0.37–0.56]), and during school time (odds ratio (odds ratio, 0.86 [95% CI, 0.77–0.96]) even when adjusted for sex, socioeconomic status, and country. A stronger association was observed in adolescents from South and Central America and Eastern Mediterranean regions in the domain of weekends and weekdays. The results indicate that early internet exposure is associated with heavy Internet use, particularly during weekends and weekdays, regardless the geographical region, in a linear fashion. Further research should aim to examine if better education and parental control in specific areas may avoid excessive Internet use that possibly have a negative influence on both mental and physical health.

**Supplementary Information:**

The online version contains supplementary material available at 10.1007/s00787-021-01869-5.

## Introduction

Internet use has been incorporated into almost every facet of life. For example, the internet is used for communication via emails, video calls, and instant messages; for entertainment, such as gaming, streaming TV shows, and music; and for work/education by providing a source of instant knowledge and information searching. The present generation of adolescents has simultaneously aged with the introduction and expansion of tablets and smartphones and the rise of social networks, which comprises activities, such as communicating, gaming, listening to music, or searching information, among others. The time US adolescents go online more than doubled between 2006 and 2016, and similar figures have been observed in other Western countries [1]. In view of this, a plethora of studies have investigated excessive internet use in adolescents, observing a predominant link to individual factors, such as gender, self-control, social anxiety or self-esteem among others, and prevalence ranging up to a maximum of 26% [2, 3].

Worryingly, detrimental effects of excessive Internet use have been associated with various detrimental health outcomes observed in adolescents; a longitudinal study by Lam et al. including adolescents initially free of mental health complications identified pathological use of the Internet as a risk factor for depression [4]. Also, a more recent study involving British adolescents showed excessive use of Internet associated with conduct problems, hyperactivity, poorer physical health, depression, and impact on daily life activities [5]. Moreover, a dose–time–response association between the use of social media and an increase in symptoms of depression, conduct problems, and episodic heavy drinking has been previously identified [6]. It should also be noted however that other studies have found limited evidence of adverse psychological effects from Internet usage in general [7], and causal mechanisms of the observed associations so far have yet to be established.

Nonetheless, there is also growing evidence of an association between intensive Internet use and worse physical health outcomes; Cassidy-Bushrow et al. [8] observed heavy internet usage (i.e., ≥ 2 h/day) in adolescents associated with significantly higher odds for elevated blood pressure compared to light Internet users. Furthermore, Internet use for more than 2 h at weekends was the only explanatory variable remaining for overweight after 2-year follow-up [9]. Moreover, Internet use for non-study reasons has been identified as an important reason for increased sedentary behaviour in European adolescent girls, which in turn can favour obesity and other related conditions [10].

Given this background, it seems of growing importance to investigate which factors might lead to heavy Internet use in different domains. One potential variable that deserves closer examination is age of first exposure, since age of initiation has been associated with problematic use of Internet in elementary school children and Internet gaming disorder [11, 12]. Currently however, there is little evidence concerning whether an early exposure might be associated with heavy Internet use in adolescence. Indeed, there is a lack of studies examining the consistence of the aforementioned association in different domains in large samples worldwide. Therefore, this study aims to investigate the association between age of first exposure and heavy Internet use in a large, representative, and widespread sample of adolescents from 52 countries.

We hypothesised that an inverse association will be observed between age of first exposure and heavy use of the Internet in adolescents, with potential differences concerning domains and geographical regions.

## Methods

### Study population and data source

The Organisation for Economic Co-operation and Development (OECD) systematically conducts a Programme for International Students Assessment (PISA) every 3 years since 2000. A total of 79 countries, including OECD and non-OECD members, participated in the latest round of PISA in 2018. Of those, only countries implementing both the Student Questionnaire, which was compulsory in the PISA 2018 main survey, and the optional Familiarity Questionnaire, which contained the main examined variables, were included in the present study (i.e., 52 countries overall). This comprised an in-school representative population of students aged between 15 years and 3 months and 16 years and 2 months at the time of assessment, or a one-month variation of this. Except for Russia, where a three-stage design first considering geographical regions was used, PISA was based on a two-stage probabilistic, stratified, and clustered survey design for the participating countries. First, schools were stratified and then a minimum of 150 schools with probability proportional to size were randomly selected from within each country. The second-stage sampling was aimed at students within sampled schools; from each list of eligible students within a school, either a sample of 42 or 35 students were selected with equal probability for computer and paper-based assessments, and for lists with fewer students than that target number, all students on the list were selected. Included countries aimed for a response rate of 85% for schools and 80% for students. Further details of both the complex survey and sampling design are available in the 2018 Technical Report [13].

Data retrieved from the public anonymized dataset derived from PISA questionnaires were used for the present study. Participants with complete values in all the variables used in this study (84%) were included in the analyses.

Countries were grouped into seven categories according to the World Health Organization (WHO) regions and sub-regions. Sub-regions are denoted by A, B and C suffices, which corresponds to a declining country-level wealth [14]. The seven categories used were the following: (1) Americas A (United States); (2) Americas B/C; (3) Eastern Mediterranean; (4) Europe A; (5) Europe B/C; (6) South-East Asian and Western Pacific; and (7) Africa (Morocco). Southeast Asian and Western Pacific regions were combined since Thailand was the only PISA participant in the former. Although Chinese Taipei and Hong Kong are not currently recognized as WHO member states, they were included into the Southeast Asian and Western Pacific region according to geography.

The study received the approval of the Ethics Committee of Research in Humans of the institution (register code 1510464), and adhered to the STROBE Statement recommendations [15].

### Age of first exposure to Internet

The exposure variable was self-reported by participants through the following question: “How old were you when you first accessed the Internet?” Answers comprised the following options: “3 years old or younger”, “4–6 years old”, “7–9 years old”, “10–12 years old”, “13 years old or older”, and “I have never accessed the Internet”. To attenuate the possibility of a recall bias for those reporting earlier exposure ages, this variable was later categorized into those having initiated the Internet at age 9 or lower, from 10 to 12 years, and 13 years or over.

### Heavy Internet use

The outcome variable was self-reported by participants through the following questions: “During a typical weekday, for how long do you use the Internet at school?”, “During a typical weekday, for how long do you use the Internet outside of school?”, “On a typical weekend day, for how long do you use the Internet outside of school?” Potential answers comprised “No time”, “1–30 min per day”, “31–60 min per day”, “Between 1 and 2 h per day”, “Between 2 and 4 h per day”, “Between 4 and 6 h per day”, and “More than 6 h per day”. According to prior research, use of the Internet was categorized into those who used it from no time to 2 h, and those who used from more than 2 h (heavy Internet use) in each of the three examined domains (i.e., at school, during weekdays, and during weekends) [16].

### Socioeconomic status

Prior research has identified inverse associations between Internet use and socioeconomic status (SES), and thus SES was incorporated into statistical models as a control variable [17]. Reported family wealth possessions, a continuous variable estimated using OECD item response theory scaling including nine standardised questions about possessions in and characteristics of the home, served to estimate SES. These included questions on whether the student had their own room, whether the home had a link to the Internet, the number of rooms in the home with a bath or shower, the number of televisions, computers, tablets, cell phones, and e-book readers in the home, and the number of cars the family has. Quintiles of this variable were calculated to use in our analyses.

### Statistics

Statistical analyses were conducted with Stata 16.1 (Stata Corp LP, College station, Texas). Multivariable logistic regression analysis was performed to assess the association between age of first exposure to Internet (exposure variable) and heavy Internet use in three different domains (outcome variables). Survey-adjusted analyses using weighted sample (eTable 1 in the Supplement) overall, as well as stratified by geographical region were conducted (eTable 2). These analyses were adjusted for sex, SES, and country. To assess the generalizability of the findings based on the overall sample and regional sub-samples, we also conducted country-wise regression analyses for the association between age of first exposure to Internet and heavy Internet use adjusted for sex and SES. Pooled estimates for each age category were obtained by metanalyses with random effects based on region-wise estimates. To assess the level of heterogeneity among regions in the examined association we also calculated the Higgins’s *I*^2^. All variables were included in the models as either continuous (SES) or categorical variables. Results from the logistic regression analyses are presented as odds ratios (ORs) with 95% confidence intervals (CIs). Sensitivity analyses examining the association stratified for sex, SES, and country using the original categories for age of first exposure to Internet were performed to check the robustness of the findings (eTable 3, eTable 4, eTable 5). Additionally, the aforementioned association was also examined using the final adjusted model with the original categories of age of first exposure to Internet and heavy use of the Internet variables using linear regression for the last case.

## Results

A total of 317,443 adolescents aged 15 and 16 years from 52 countries constituted the final sample, of whom 161,148 were girls (50.8%). Data on heavy Internet use during weekdays, weekends, and school time, as well as SES and first exposure to Internet use are reported in Table 1. The majority of students reported Internet exposure at ≤ 9 years old (60.3%).

**Table 1:** Sample characteristics (overall and by heavy Internet use) (*N* = 317,443)

Characteristic	Category	Overall	Heavy Internet use during weekdays	Heavy Internet use during weekends	Heavy Internet use in the school
Age of first exposure to Internet	9 years or lower	60.3	71.8	81.6	25.0
	10–12 years	30.7	63.1	74.0	20.6
	13 years or over	9.0	47.6	57.5	19.2
Sex	Boys	49.2	65.5	75.6	22.6
	Girls	50.8	68.3	78.5	23.7
Region	Americas A	1.3	77.5	82.6	38.6
	Americas B/C	11.6	66.2	72.0	21.8
	Eastern Mediterranean	7.8	62.7	72.8	17.9
	Europe A	32.9	73.0	82.9	23.0
	Europe B/C	25.0	64.2	73.3	24.7
	Southeast Asian and Western Pacific	19.9	64.2	78.8	24.0
	Africa	1.5	30.5	44.8	13.5
Socioeconomic status	First quintile	15.3	50.7	61.3	18.0
	Second quintile	16.1	63.9	74.8	20.2
	Third quintile	20.5	67.4	78.2	21.6
	Fourth quintile	20.1	70.7	80.8	23.7
	Fifth quintile	28.0	74.3	83.4	28.4

In total, 212,395 (66.9%) and 244,621 (77.1%) adolescents, respectively, reported heavy Internet use during weekdays and weekends, excluding school time. In addition, 73,474 (23.2%) adolescents reported heavy Internet use in school. Information on prevalence of heavy Internet use for each country is shown in Table 2. The highest prevalence of heavy Internet use during weekdays (85.4%) and weekends (91.8%) was observed in Sweden, whereas the highest prevalence for heavy Internet use in school was observed in Denmark (70.5%).

**Table 2:** Sample size and prevalence of heavy Internet use by country

Geographical areas	Country	*N*	Female (%)	Heavy Internet use during weekdays (%)	Heavy Internet use during weekends (%)	Heavy Internet use in the school (%)
	Overall	317,443	50.8	66.9	77.1	23.2
Americas A	United States	4081	49.9	77.5	82.6	38.6
Americas B/C	Brazil	7450	52.6	71.6	76.6	14.6
	Chile	6016	49.8	77.5	83.9	32.1
	Costa Rica	5887	50.8	72.5	77.2	31.7
	Dominican Republic	4063	51.8	53.2	59.2	14.1
	Mexico	5792	52.3	57.3	60.7	17.4
	Panama	4264	49.6	48.7	62.8	11.0
	Uruguay	3328	53.8	76.6	78.0	32.7
Eastern Mediterranean	Albania	5295	50.4	47.4	59.6	10.1
	Croatia	5879	51.6	69.7	82.1	22.9
	Malta	2764	52.5	76.8	83.0	10.9
	Serbia	4909	51.6	74.4	79.2	23.4
	Turkey	6125	49.7	53.5	65.6	18.5
Europe A	Austria	6009	50.6	68.9	76.5	23.5
	Belgium	7042	51.8	75.1	87.2	16.8
	Denmark	6280	51.5	81.5	89.7	70.5
	Finland	4956	50.5	75.1	85.6	25.8
	France	5411	49.7	68.6	83.3	16.7
	Greece	5466	51.5	65.4	81.0	20.5
	Iceland	2754	52.3	74.2	84.5	30.9
	Ireland	5062	50.6	74.8	85.6	9.8
	Italy	10,024	49.4	70.2	71.0	19.7
	Luxembourg	4440	49.1	73.9	82.9	22.9
	Spain	30,448	50.7	71.6	83.4	16.5
	Sweden	4580	51.9	85.4	91.8	52.1
	Switzerland	5035	48.5	64.4	76.0	17.5
	United Kingdom	6787	51.5	81.2	89.4	15.0
Europe B/C	Bulgaria	3638	50.8	71.5	76.0	36.0
	Czech Republic	6124	50.7	62.1	71.7	16.3
	Estonia	4770	51.2	72.1	79.4	28.5
	Georgia	3972	51.3	60.6	69.0	11.5
	Hungary	4687	51.3	71.7	82.1	23.5
	Kazakhstan	17,084	50.1	50.7	63.3	24.3
	Latvia	4589	51.4	73.8	79.5	28.1
	Lithuania	6082	50.3	73.9	78.4	23.5
	Poland	5108	51.8	72.9	82.6	23.7
	Russian Federation	6341	52.1	67.2	76.3	28.4
	Slovak Republic	4983	51.2	67.1	73.7	26.9
	Slovenia	5496	48.1	59.1	70.9	21.3
	Moscow Region (RUS)	1735	49.5	69.1	77.8	32.0
	Tatarstan (RUS)	4715	52.1	64.4	72.5	30.7
South-East Asian and Western Pacific	Australia	10.922	50.1	76.7	83.2	47.1
	Brunei Darussalam	5590	50.8	68.1	71.3	6.8
	Chinese Taipei	6499	50.0	55.7	78.4	20.1
	Hong Kong	5401	50.3	66.2	78.9	8.7
	Japan	5625	51.0	48.1	73.4	7.7
	Korea	6283	48.4	43.3	69.0	8.2
	Macao	3331	50.4	66.9	83.5	10.6
	New Zealand	5474	52.2	77.8	83.5	48.0
	Singapore	6168	49.0	73.1	84.3	21.0
	Thailand	7943	54.7	60.7	80.3	33.7
Africa	Morocco	4696	47.0	30.5	44.8	13.5

**Fig. 1:**
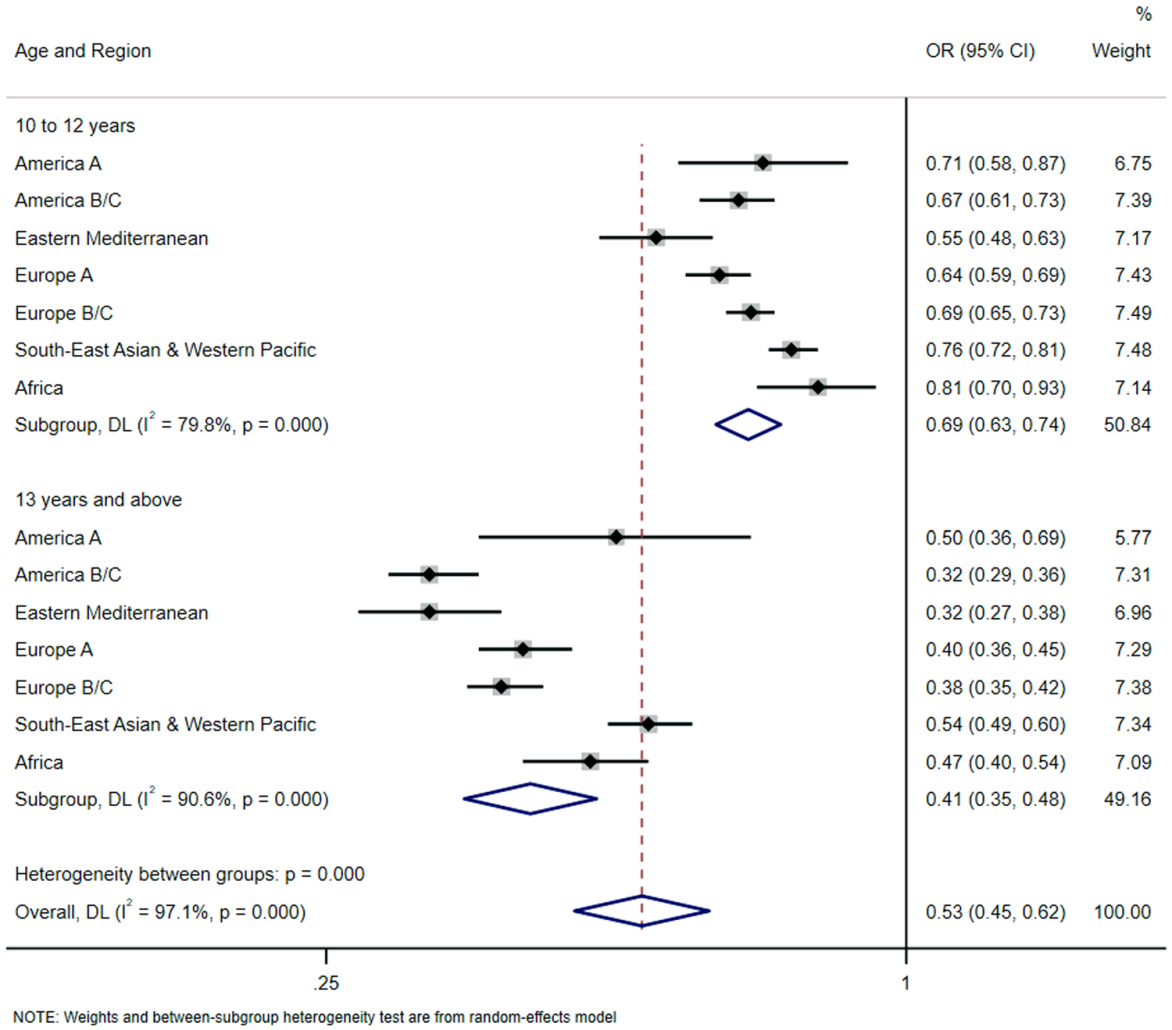
Regional-wise associations between age of first exposure to Internet (exposure) and heavy Internet use during weekend (outcome). *OR* odds ratio, *CI* confidence interval. Overall estimate was obtained by meta-analysis with random effects. The model is survey-adjusted, weighted, and additionally adjusted for sex, country, and socioeconomic status

Figure 1 displays overall and region-wise estimates for heavy Internet use during weekends. Overall, exposure to Internet at 10 years old or over was associated with significantly lower odds for heavy Internet use during weekends at 15 or 16 years old (odds ratio, 0.53 [95% CI, 0.45–0.62]). Subgroup of adolescents reporting age of Internet initiation at ≥ 13 years showed the highest inverse association (odds ratio, 0.41 [95% CI, 0.35–0.48]), which was more accentuated in countries from America B/C (odds ratio, 0.32 [95% CI, 0.29–0.36]), and Eastern Mediterranean region (odds ratio, 0.32 [95% CI, 0.27–0.38]).

**Fig. 2:**
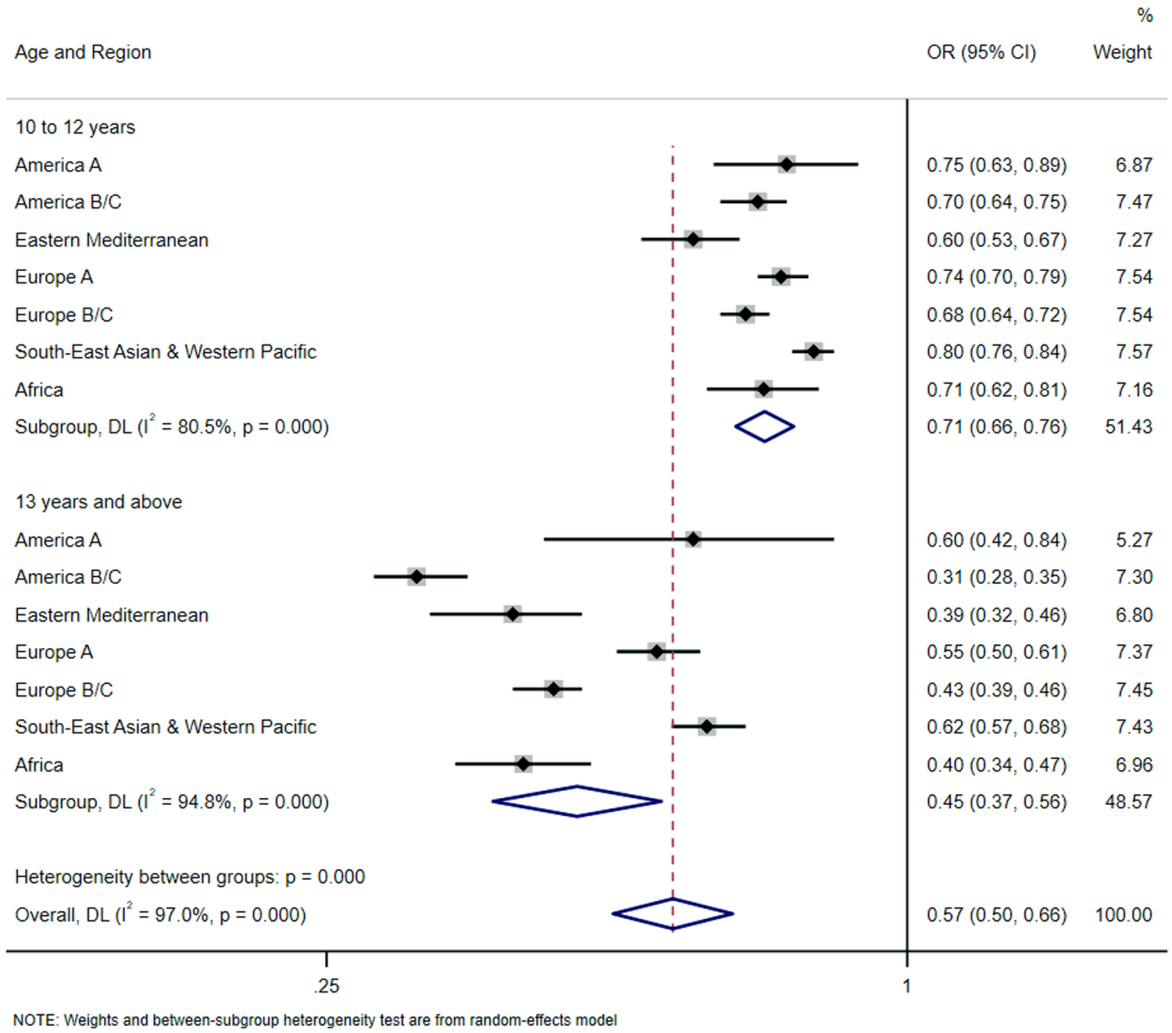
Regional-wise associations between age of first exposure to Internet (exposure) and heavy Internet use during weekdays excluding school (outcome). *OR* odds ratio, *CI* confidence interval. Overall estimate was obtained by meta-analysis with random effects. The model is survey-adjusted, weighted, and additionally adjusted for sex, country, and socioeconomic status

Regarding heavy Internet use during weekdays (Fig. 2), overall values for the subgroup of adolescents whose first Internet exposure age was ≥ 10 years, showed a significant lower odds ratio (0.57 [95% CI, 0.50–0.66]). The subgroup of adolescents aged 13 years and above showed lower odds ratio (0.45 [95% CI, 0.37–0.56]), with countries from the America B/C region displaying the lowest values (odds ratio, 0.31 [95% CI, 0.28–0.35]).

**Fig. 3:**
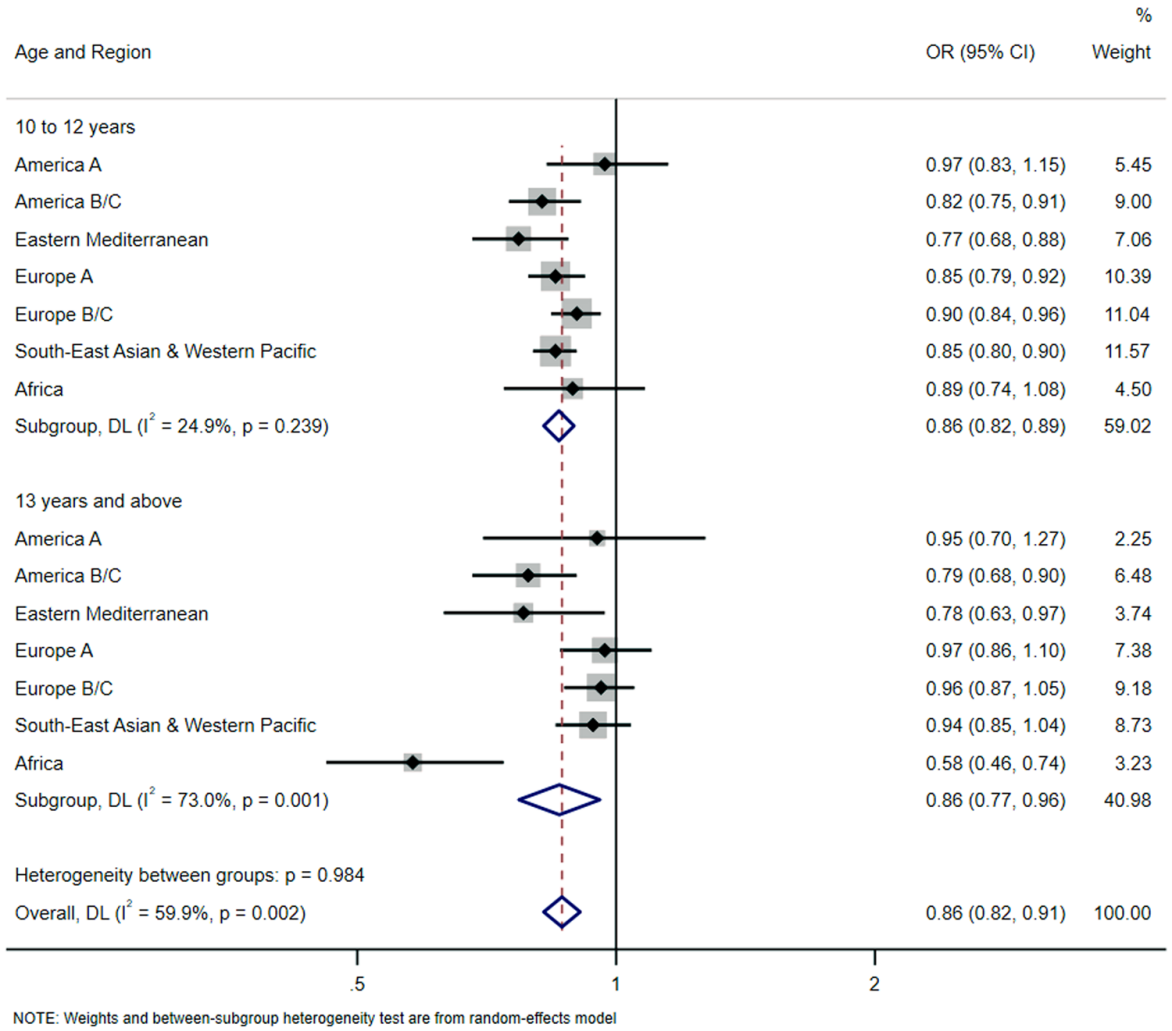
Regional-wise associations between age of first exposure to Internet (exposure) and heavy Internet use during school (outcome). *OR* odds ratio, *CI* confidence interval. Overall estimate was obtained by meta-analysis with random effects. The model is survey-adjusted, weighted, and additionally adjusted for sex, country, and socioeconomic status

Figure 3 presents estimates concerning heavy Internet use in school. Overall values showed lower significant odds (odds ratio, 0.86 [95% CI, 0.82–0.91]), with similar values for the 10–12 years old (odds ratio, 0.86 [95% CI, 0.82–0.89]), and the 13 years and above subgroup (odds ratio, 0.86 [95% CI, 0.77–0.96]). African adolescents whose age of first exposure to Internet use was 13 years or above showed the lowest odds for heavy use of the Internet in the school (odds ratio, 0.58 [95% CI, 0.46–0.74]).

Sensitivity analyses showed similar results for the examined association using the original age of first exposure to Internet categories as well as for the original categories for heavy use of the Internet in the three domains.

## Discussion

To our knowledge, this is the first study analysing age of Internet initiation and heavy Internet use among adolescents from a large international sample. The prevalence of heavy Internet use during both weekends and weekdays outside schools remained consistently higher for those adolescents with an earlier age of first exposure to the Internet, in a significant linear fashion in the examined geographical regions. However, these trends were not completely confirmed for less represented geographical areas, such as Africa and Americas A, with regard to school time internet usage. Prevalence of heavy Internet use was substantially higher during weekends and weekdays in relation to school time, which also showed significant higher odds of heavy use of the Internet overall for those whose first Internet exposure age was ≤ 9 years old. In particular, both Eastern Mediterranean and America B/C regions pointed at a stronger negative association between earlier age of first exposure and heavy Internet use during weekends and weekdays outside of school, and during school time, although the latter was importantly less accentuated. These results support the notion of a widespread potential link between age of first exposure and heavy Internet use during adolescence. Within this, the findings further indicate that these associations vary with regards to the examined domain (i.e., the association is more pronounced during weekends and weekdays outside the school than during school time), and the geographical region.

A study by Martins et al. [18] observed a negative association between Internet addiction in adolescents and parental control. Hence, parental control may play a critical role when modulating the use of Internet among adolescents and could help to explain the lower prevalence found for heavy Internet use during school time, as schools are usually supervised environments which could persuade adolescents away from an indiscriminate use of the Internet. Furthermore, because parental control has been observed to vary among countries, this could partly explain geographical differences found in the present study. In particular, parental control has been observed to be higher in Eastern than Western countries [19] which is consistent with the higher prevalence of heavy Internet use during weekend and weekdays we found for America A and Europe A geographical areas. Moreover, since parental awareness regarding Internet use of adolescents has been found higher in mothers than fathers [20], it is plausible that those countries with higher prevalence of mothers involved in parental control of children present lower heavy use of the Internet in adolescents.

In addition, the use of Internet for educational purposes (i.e., assignments requiring the use of Internet) has been previously observed to increase overall Internet use in high-school students. Therefore, countries with educational systems tending to higher number of assignments requiring the use of Internet may be more likely to have higher prevalence of heavy Internet use among their adolescent-schooled population [21]. In view of this, it would be plausible to consider those countries with higher accessibility to Internet as more prone to use the Internet in their educational schemes which, in turn, can importantly increase heavy use of the Internet among adolescents. This argument is coherent with the high prevalence of heavy Internet use of adolescents during school time and outside school at weekends and weekdays observed in this study for very high-income countries with supposedly full access to the Internet, such as Sweden or Denmark. On the other hand, the higher prevalence of heavy Internet use we found at weekends endorses the findings of previous research, which observed higher prevalence of sedentary behaviours in European adolescents usually associated with Internet use for not academic purposes [10].

Regarding the observed association between age of first exposure and heavy Internet use in adolescence, prior research observed more serious problematic Internet use in elementary school among Taiwanese children exhibiting earlier experiences of using the Internet [12]. This strengthens the possibility of an association between the two aforementioned study variables that might go back earlier than the age of 15 or 16 years. As it has been observed for younger children, those adolescents going online through more types of devices, might also have more serious problems with Internet use [12]. Nevertheless, our study observed a consistent negative association between age of use of initiation and heavy Internet use regardless of SES (defined in this study by the ownership of a number of items, including different digital devices) which indicates that the association is robust to this confounder. Similarly, sex does not seem to substantially modify our results since both adjusted and sensitivity analyses involving sex continue showing a strong association. By contrast, a Korean study observed the association between Internet addiction and adverse psychological health outcomes was more pronounced in boys than in girls [22], although the different outcomes used make comparisons with our study difficult.

The results of this study should be interpreted in the light of several limitations, including the possibility of a recall bias in the cross-sectional data, although the impact of this may have been attenuated through the large representative sample study used. Additionally, owing to the high percentage of missing participants from specific countries, there is still the possibility of a selection bias, although the widespread sample across 52 countries from 7 different geographical areas is enough to confirm the observed association. However, we found a higher heterogeneity among categories of age for first exposure to Internet in the different geographical regions, which might strengthen the notion that differences regarding geographical regions are critical. Moreover, the scope of the findings is limited, and does not allow to analyse whether there is any type of digital device, content, or an accurate year of initiation associated with higher consumption of the Internet. Further research might discriminate usage that can be detrimental for health from usage that can be health-enhancing, such as specific exposures to particular social networks that may improve relevant dimensions of adolescents’ self-construction [23]. Also, because there is little information regarding either low-income or African countries, generalizations over populations from countries with such features should be cautiously made. Moreover, because Africa and Americas A regions are substantially less represented in the study (i.e., lower number of participants from a single country), results of these regions should be interpreted with caution. Finally, because the current COVID-19 pandemic is affecting lifestyles worldwide, habits concerning Internet will have likely changed over this period, probably increasing the use of Internet among adolescents [24].

## Conclusion

Adolescents whose age of first exposure to Internet was ≤ 9 years exhibited significant higher odds for heavy use of the Internet during weekends, weekdays, and school time, although the latter was weaker. This association was stronger in adolescents from America B/C and Eastern Mediterranean countries, who might experience less parental control outside school than those from other geographical regions. To avoid excessive Internet usage as well as to inform preventive strategies, future research examining the causes underpinning the association between age of first exposure and heavy use of the Internet is warranted.

## Supplementary Information

Below is the link to the electronic supplementary material.

Supplementary file1 (DOCX 35 kb)

## Data Availability

The technical report and the 2018 PISA dataset used in this study are publicly available at https://www.oecd.org/pisa/data/.
