# Supplementary material for: Association between age of first exposure and heavy internet use in a representative sample of 317,443 adolescents from 52 countries
Source: Eur Child Adolesc Psychiatry. 2021 Sep 12;32(3):395–403. doi: 10.1007/s00787-021-01869-5 (PMC8435168; doi:10.1007/s00787-021-01869-5)
Supplement: Supplementary file 1 — Supplementary file1 (DOCX 35 kb) [file 787_2021_1869_MOESM1_ESM.docx]

| **eTable 1. Weighted sample.** | | |
| --- | --- | --- |
| Region | Country | Weighted N |
| America A | United States | 3,559,045 |
| America B/C | Brazil | 2,036,861 |
|  | Chile | 213,832 |
|  | Costa rica | 45,475 |
|  | Dominican Republic | 140,330 |
|  | Mexico | 1,480,904 |
|  | Panama | 38,540 |
|  | Uruguay | 39,746 |
| Eastern Mediterranean | Albania | 27,963 |
|  | Croatia | 35,462 |
|  | Malta | 3,925 |
|  | Serbia | 61,895 |
|  | Turkey | 884,971 |
| Europe A | Austria | 75,077 |
|  | Belgium | 118,025 |
|  | Denmark | 59,967 |
|  | Finland | 56172 |
|  | France | 756,477 |
|  | Greece | 95,370 |
|  | Iceland | 3,878 |
|  | Ireland | 59,639 |
|  | Italy | 521,223 |
|  | Luxembourg | 5,478 |
|  | Spain | 416,703 |
|  | Sweden | 93,129 |
|  | Switzerland | 71,683 |
|  | United Kingdom | 3,559,045 |
| Europe B/C | Bulgaria | 47,851 |
|  | Czech Republic | 87,808 |
|  | Estonia | 11,415 |
|  | Georgia | 38,489 |
|  | Hungary | 86,754 |
|  | Kazakhstan | 212,229 |
|  | Latvia | 15,932 |
|  | Lithuania | 24,453 |
|  | Poland | 318,724 |
|  | Russian Federation | 1,257,388 |
|  | Slovak Republic | 44,418 |
|  | Slovenia | 17,138 |
|  | Moscow Region (RUS) | 1,257,388 |
|  | Tatarstan (RUS) | 1,257,388 |
| South-East Asian & Western Pacific | Australia | 257,779 |
|  | Brunei Darussalam | 6,899 |
|  | Chinese Taipei | 226,698 |
|  | Hong Kong | 51,101 |
|  | Japan | 1,078,921 |
|  | Korea | 455,544 |
|  | Macao | 3,799 |
|  | New Zealand | 53,000 |
|  | Singapore | 44,058 |
|  | Thailand | 575,713 |
| Africa | Morocco | 386,408 |

| **eTable 2. Country-wise association between age of first exposure and heavy Internet use during adolescence adjusted for control variables.** | | | | | | | | | | |
| --- | --- | --- | --- | --- | --- | --- | --- | --- | --- | --- |
| Geographical areas | Country | Heavy Internet use weekdays (OR) (95% CI) | | | Heavy Internet use during weekends (OR) (95% CI) | | | Heavy Internet use in the school (OR) (95% CI) | | |
|  |  | 9 years or lower | 10 to 12 years | 13 years or over | 9 years or lower | 10 to 12 years | 13 years or over | 9 years or lower | 10 to 12 years | 13 years or over |
|  | Overall | ref | 0.65 (0.63-0.68) | 0.35 (0.33-0.37) | ref | 0.70 (0.67-0.72) | 0.41 (0.39-0.43) | ref | 0.89 (0.85-0.92) | 0.91 (0.86-0.96) |
| America A | United States | ref | 0.75 (0.63-0.89) | 0.6 (0.42-0.84) | ref | 0.71 (0.58-0.87) | 0.5 (0.36-0.69) | ref | 0.97 (0.83-1.15) | 0.95 (0.7-1.27) |
| America B/C | Brazil | ref | 0.74 (0.66-0.84) | 0.3 (0.26-0.35) | ref | 0.67 (0.59-0.76) | 0.33 (0.28-0.38) | ref | 0.79 (0.68-0.92) | 0.92 (0.74-1.13) |
|  | Chile | ref | 0.72 (0.63-0.83) | 0.49 (0.39-0.61) | ref | 0.71 (0.6-0.85) | 0.39 (0.31-0.5) | ref | 0.91 (0.8-1.04) | 0.78 (0.62-0.98) |
|  | Costa Rica | ref | 0.71 (0.63-0.81) | 0.37 (0.31-0.44) | ref | 0.67 (0.59-0.76) | 0.36 (0.3-0.43) | ref | 0.66 (0.57-0.75) | 0.53 (0.42-0.67) |
|  | Dominican Republic | ref | 0.83 (0.69-0.99) | 0.41 (0.34-0.49) | ref | 0.84 (0.7-1) | 0.47 (0.39-0.57) | ref | 0.84 (0.67-1.06) | 0.79 (0.61-1.01) |
|  | Mexico | ref | 0.64 (0.56-0.72) | 0.32 (0.27-0.39) | ref | 0.67 (0.58-0.76) | 0.32 (0.27-0.38) | ref | 0.84 (0.71-0.99) | 0.66 (0.53-0.82) |
|  | Panama | ref | 0.7 (0.6-0.81) | 0.37 (0.31-0.44) | ref | 0.76 (0.65-0.89) | 0.42 (0.36-0.5) | ref | 1.05 (0.83-1.32) | 1.07 (0.82-1.42) |
|  | Uruguay | ref | 0.76 (0.63-0.92) | 0.47 (0.33-0.67) | ref | 0.83 (0.67-1.03) | 0.41 (0.3-0.56) | ref | 1.01 (0.82-1.24) | 1.24 (0.88-1.73) |
| Eastern Mediterranean | Albania | ref | 0.74 (0.65-0.85) | 0.45 (0.38-0.53) | ref | 0.84 (0.74-0.96) | 0.5 (0.43-0.58) | ref | 0.76 (0.62-0.93) | 0.93 (0.71-1.21) |
|  | Croatia | ref | 0.72 (0.63-0.82) | 0.48 (0.37-0.62) | ref | 0.72 (0.61-0.85) | 0.42 (0.33-0.54) | ref | 0.79 (0.69-0.91) | 0.69 (0.5-0.95) |
|  | Malta | ref | 0.61 (0.48-0.77) | 0.42 (0.28-0.64) | ref | 0.65 (0.52-0.81) | 0.3 (0.2-0.46) | ref | 1.09 (0.79-1.5) | 3.5 (2.28-5.38) |
|  | Serbia | ref | 0.77 (0.67-0.89) | 0.51 (0.4-0.65) | ref | 0.8 (0.69-0.94) | 0.45 (0.34-0.6) | ref | 0.84 (0.72-0.98) | 1.21 (0.95-1.53) |
|  | Turkey | ref | 0.58 (0.52-0.66) | 0.39 (0.32-0.47) | ref | 0.53 (0.46-0.61) | 0.31 (0.26-0.38) | ref | 0.77 (0.66-0.89) | 0.78 (0.62-0.99) |
| Europe A | Austria | ref | 0.76 (0.67-0.86) | 0.57 (0.45-0.72) | ref | 0.81 (0.71-0.93) | 0.44 (0.34-0.56) | ref | 0.94 (0.83-1.06) | 1.09 (0.81-1.46) |
|  | Belgium | ref | 0.84 (0.74-0.94) | 0.55 (0.45-0.68) | ref | 0.84 (0.71-0.98) | 0.5 (0.4-0.62) | ref | 0.87 (0.77-1) | 1.28 (1.01-1.62) |
|  | Denmark | ref | 0.81 (0.69-0.96) | 0.35 (0.22-0.57) | ref | 0.7 (0.58-0.84) | 0.43 (0.25-0.76) | ref | 0.75 (0.65-0.88) | 0.51 (0.32-0.82) |
|  | Finland | ref | 0.74 (0.63-0.87) | 0.79 (0.38-1.64) | ref | 0.69 (0.56-0.85) | 0.34 (0.17-0.68) | ref | 0.86 (0.71-1.04) | 0.97 (0.44-2.13) |
|  | France | ref | 0.71 (0.63-0.8) | 0.56 (0.45-0.68) | ref | 0.57 (0.49-0.67) | 0.35 (0.28-0.44) | ref | 0.88 (0.74-1.04) | 1.2 (0.9-1.59) |
|  | Greece | ref | 0.8 (0.71-0.89) | 0.61 (0.5-0.73) | ref | 0.74 (0.64-0.85) | 0.46 (0.37-0.56) | ref | 0.97 (0.83-1.12) | 1.41 (1.13-1.76) |
|  | Iceland | ref | 0.72 (0.59-0.88) | 1.11 (0.57-2.17) | ref | 0.64 (0.5-0.81) | 0.82 (0.39-1.71) | ref | 0.7 (0.58-0.86) | 1.36 (0.8-2.33) |
|  | Ireland | ref | 0.62 (0.53-0.72) | 0.4 (0.31-0.5) | ref | 0.66 (0.56-0.79) | 0.29 (0.23-0.38) | ref | 0.76 (0.62-0.94) | 0.95 (0.68-1.33) |
|  | Italy | ref | 0.78 (0.71-0.86) | 0.63 (0.55-0.72) | ref | 0.77 (0.7-0.84) | 0.58 (0.5-0.67) | ref | 0.77 (0.7-0.86) | 0.84 (0.72-0.98) |
|  | Luxembourg | ref | 0.73 (0.63-0.85) | 0.63 (0.49-0.8) | ref | 0.74 (0.62-0.9) | 0.43 (0.33-0.55) | ref | 0.86 (0.72-1.03) | 0.92 (0.66-1.29) |
|  | Spain | ref | 0.79 (0.75-0.84) | 0.56 (0.5-0.62) | ref | 0.74 (0.69-0.79) | 0.5 (0.44-0.57) | ref | 0.92 (0.85-0.99) | 1.06 (0.91-1.23) |
|  | Sweden | ref | 0.66 (0.55-0.8) | 0.35 (0.23-0.54) | ref | 0.74 (0.56-0.96) | 0.44 (0.27-0.72) | ref | 0.78 (0.67-0.9) | 0.96 (0.66-1.41) |
|  | Switzerland | ref | 0.72 (0.63-0.82) | 0.64 (0.52-0.78) | ref | 0.67 (0.59-0.77) | 0.52 (0.42-0.65) | ref | 0.94 (0.81-1.09) | 1 (0.77-1.3) |
|  | United Kingdom | ref | 0.75 (0.65-0.86) | 0.55 (0.4-0.74) | ref | 0.64 (0.53-0.79) | 0.39 (0.27-0.55) | ref | 0.88 (0.74-1.05) | 0.98 (0.66-1.45) |
| Europe B/C | Bulgaria | ref | 0.73 (0.62-0.87) | 0.49 (0.35-0.68) | ref | 0.79 (0.64-0.97) | 0.54 (0.39-0.74) | ref | 0.77 (0.65-0.91) | 1.17 (0.86-1.6) |
|  | Czech Republic | ref | 0.77 (0.69-0.86) | 0.75 (0.58-0.96) | ref | 0.75 (0.67-0.84) | 0.58 (0.45-0.76) | ref | 0.92 (0.78-1.09) | 1.28 (0.94-1.74) |
|  | Estonia | ref | 0.7 (0.57-0.85) | 0.62 (0.4-0.97) | ref | 0.69 (0.58-0.82) | 0.46 (0.29-0.74) | ref | 0.92 (0.76-1.12) | 1.26 (0.83-1.9) |
|  | Georgia | ref | 0.71 (0.61-0.83) | 0.48 (0.38-0.6) | ref | 0.68 (0.57-0.8) | 0.36 (0.28-0.47) | ref | 0.94 (0.73-1.21) | 1.61 (1.16-2.23) |
|  | Hungary | ref | 0.75 (0.65-0.85) | 0.48 (0.38-0.62) | ref | 0.75 (0.64-0.87) | 0.32 (0.25-0.41) | ref | 0.93 (0.79-1.11) | 1.18 (0.88-1.58) |
|  | Kazakhstan | ref | 0.73 (0.68-0.79) | 0.42 (0.38-0.45) | ref | 0.75 (0.69-0.81) | 0.4 (0.36-0.44) | ref | 0.78 (0.72-0.85) | 0.66 (0.6-0.73) |
|  | Latvia | ref | 0.77 (0.65-0.91) | 0.59 (0.43-0.82) | ref | 0.72 (0.6-0.85) | 0.6 (0.42-0.86) | ref | 0.87 (0.73-1.04) | 1.07 (0.77-1.47) |
|  | Lithuania | ref | 0.78 (0.67-0.91) | 0.43 (0.32-0.56) | ref | 0.67 (0.57-0.78) | 0.41 (0.3-0.55) | ref | 1.03 (0.88-1.21) | 1.26 (0.9-1.77) |
|  | Poland | ref | 0.65 (0.53-0.81) | 0.48 (0.33-0.68) | ref | 0.67 (0.57-0.8) | 0.63 (0.45-0.89) | ref | 0.85 (0.72-0.99) | 1.07 (0.77-1.5) |
|  | Russian Federation | ref | 0.76 (0.65-0.89) | 0.54 (0.39-0.75) | ref | 0.65 (0.57-0.74) | 0.33 (0.27-0.39) | ref | 0.93 (0.81-1.06) | 0.96 (0.8-1.14) |
|  | Slovak Republic | ref | 0.64 (0.57-0.72) | 0.36 (0.31-0.43) | ref | 0.83 (0.73-0.95) | 0.53 (0.42-0.66) | ref | 0.91 (0.79-1.04) | 1.22 (0.96-1.54) |
|  | Slovenia | ref | 0.83 (0.73-0.95) | 0.59 (0.48-0.74) | ref | 0.72 (0.63-0.81) | 0.48 (0.38-0.6) | ref | 0.85 (0.74-0.98) | 0.95 (0.7-1.28) |
|  | Moscow Region (RUS) | ref | 0.71 (0.63-0.8) | 0.45 (0.36-0.57) | ref | 0.54 (0.42-0.7) | 0.34 (0.23-0.52) | ref | 0.75 (0.6-0.94) | 1 (0.69-1.46) |
|  | Tatarstan (RUS) | ref | 0.73 (0.64-0.83) | 0.62 (0.51-0.76) | ref | 0.8 (0.7-0.92) | 0.54 (0.43-0.67) | ref | 0.92 (0.8-1.04) | 1.09 (0.89-1.34) |
| South-East Asian & Western Pacific | Australia | ref | 0.72 (0.65-0.8) | 0.49 (0.41-0.59) | ref | 0.7 (0.63-0.78) | 0.43 (0.36-0.51) | ref | 0.81 (0.74-0.88) | 0.68 (0.57-0.82) |
|  | Brunei Darussalam | ref | 0.82 (0.71-0.95) | 0.53 (0.45-0.62) | ref | 0.75 (0.64-0.87) | 0.47 (0.39-0.56) | ref | 0.86 (0.62-1.19) | 1.06 (0.7-1.59) |
|  | Chinese Taipei | ref | 0.93 (0.84-1.03) | 0.76 (0.63-0.92) | ref | 0.85 (0.75-0.96) | 0.52 (0.42-0.64) | ref | 1.04 (0.91-1.18) | 1.35 (1.12-1.64) |
|  | Hong Kong | ref | 0.78 (0.68-0.9) | 0.48 (0.34-0.68) | ref | 0.75 (0.66-0.87) | 0.55 (0.38-0.82) | ref | 1.32 (1.08-1.62) | 2.14 (1.31-3.49) |
|  | Japan | ref | 0.74 (0.66-0.83) | 0.53 (0.44-0.64) | ref | 0.72 (0.63-0.83) | 0.56 (0.45-0.69) | ref | 0.74 (0.61-0.9) | 0.95 (0.66-1.35) |
|  | Korea | ref | 1.03 (0.93-1.15) | 1.04 (0.84-1.3) | ref | 0.89 (0.8-1) | 0.75 (0.6-0.94) | ref | 1.1 (0.91-1.34) | 1.15 (0.8-1.67) |
|  | Macao | ref | 0.68 (0.57-0.83) | 0.64 (0.45-0.92) | ref | 0.66 (0.54-0.81) | 0.53 (0.3-0.93) | ref | 0.93 (0.7-1.24) | 1.2 (0.72-2.03) |
|  | New Zealand | ref | 0.76 (0.66-0.88) | 0.42 (0.32-0.54) | ref | 0.65 (0.56-0.76) | 0.39 (0.3-0.51) | ref | 0.88 (0.78-0.99) | 0.83 (0.65-1.05) |
|  | Singapore | ref | 0.7 (0.63-0.79) | 0.53 (0.41-0.68) | ref | 0.8 (0.67-0.94) | 0.41 (0.32-0.53) | ref | 0.87 (0.74-1.02) | 1.02 (0.76-1.35) |
|  | Thailand | ref | 0.85 (0.77-0.94) | 0.61 (0.53-0.71) | ref | 0.75 (0.66-0.85) | 0.48 (0.41-0.57) | ref | 0.91 (0.81-1.02) | 0.9 (0.77-1.05) |
| Africa | Morocco | ref | 0.71 (0.61-0.81) | 0.4 (0.34-0.47) | ref | 0.81 (0.7-0.93) | 0.47 (0.4-0.54) | ref | 0.89 (0.74-1.08) | 0.58 (0.46-0.74) |
| Overall analyses adjusted for age, gender, country, and socioeconomic status.  Individual-country analyses adjusted for age, gender, and socioeconomic status. | | | | | | | | | | |

| **eTable 3. Association between age of first exposure and heavy Internet use during weekends adjusted for control variables.** | | | | | |
| --- | --- | --- | --- | --- | --- |
|  | OR (95% IC) |  |  |  |  |
|  | 3 years or less | 4-6 years | 7-9 years | 10-12 years | 13 years or older |
| Overall | ref | 1.18 (1.05-1.34) | 0.91 (0.81-1.01) | 0.57 (0.51-0.63) | 0.26 (0.23-0.30) |
| Sex | ref | 1.16 (1.03-1.32) | 0.88 (0.79-0.99) | 0.55(0.49-0.62) | 0.26 (0.23-0.29) |
| Socioeconomic status | ref | 1.23 (1.08-1.38) | 1.01 (0.90-1.13) | 0.70 (0.62-0.79) | 0.38 (0.34-0.43) |
| Country | ref | 1.19 (1.05-1.34) | 0.92 (0.82-1.03) | 0.58 (0.52-0.65) | 0.28 (0.25-0.31) |

| **eTable 4. Association between age of first exposure and heavy Internet use during weekdays adjusted for control variables.** | | | | | |
| --- | --- | --- | --- | --- | --- |
|  | OR (95% IC) |  |  |  |  |
|  | 3 years or less | 4-6 years | 7-9 years | 10-12 years | 13 years or older |
| Overall | ref | 1.00 (0.90-1.11) | 0.75 (0.67-0.83) | 0.52 (0.47-0.57) | 0.27 (0.24-0.30) |
| Sex | ref | 0.99 (0.89-1.10) | 0.73 (0.66-0.81) | 0.50 (0.45-0.56) | 0.26 (0.23-0.29) |
| Socioeconomic status | ref | 1.03 (0.92-1.15) | 0.82 (0.73-0.90) | 0.61 (0.55-0.68) | 0.36 (0.32-0.40) |
| Country | ref | 1.00 (0.90-1.12) | 0.75 (0.68-0.83) | 0.53 (0.48-0.58) | 0.28 (0.25-0.31) |

| **eTable 5. Association between age of first exposure and heavy Internet use at school adjusted for control variables.** | | | | | |
| --- | --- | --- | --- | --- | --- |
|  | OR (95% IC) |  |  |  |  |
|  | 3 years or less | 4-6 years | 7-9 years | 10-12 years | 13 years or older |
| Overall | ref | 0.71 (0.64-0.79) | 0.61 (0.55-0.70) | 0.54 (0.49-0.60) | 0.51 (0.45-0.57) |
| Sex | ref | 0.71 (0.64-0.79) | 0.60 (0.54-0.66) | 0.53 (0.48-0.59) | 0.50 (0.45-0.56) |
| Socioeconomic status | ref | 0.72 (0.65-0.80) | 0.64 (0.58-0.71) | 0.60 (0.54-0.66) | 0.61 (0.54-0.68) |
| Country | ref | 0.72 (0.65-0.80) | 0.62 (0.56-0.68) | 0.56 (0.51-0.62) | 0.54 (0.48-0.60) |
